# Finding Evidence for Local Transmission of Contagious Disease in Molecular Epidemiological Datasets

**DOI:** 10.1371/journal.pone.0069875

**Published:** 2013-07-26

**Authors:** Rolf J. F. Ypma, Tjibbe Donker, W. Marijn van Ballegooijen, Jacco Wallinga

**Affiliations:** 1 Center for Infectious Disease Control, National Institute of Public Health and the Environment, Bilthoven, The Netherlands; 2 Julius Centre for Health Sciences and Primary Care, University Medical Centre Utrecht, Utrecht, The Netherlands; 3 Medical Microbiology, University Medical Centre Groningen, Groningen, The Netherlands; University of Oxford, Viet Nam

## Abstract

Surveillance systems of contagious diseases record information on cases to monitor incidence of disease and to evaluate effectiveness of interventions. These systems focus on a well-defined population; a key question is whether observed cases are infected through local transmission within the population or whether cases are the result of importation of infection into the population. Local spread of infection calls for different intervention measures than importation of infection. Besides standardized information on time of symptom onset and location of cases, pathogen genotyping or sequencing offers essential information to address this question. Here we introduce a method that takes full advantage of both the genetic and epidemiological data to distinguish local transmission from importation of infection, by comparing inter-case distances in temporal, spatial and genetic data. Cases that are part of a local transmission chain will have shorter distances between their geographical locations, shorter durations between their times of symptom onset and shorter genetic distances between their pathogen sequences as compared to cases that are due to importation. In contrast to generic clustering algorithms, the proposed method explicitly accounts for the fact that during local transmission of a contagious disease the cases are caused by other cases. No pathogen-specific assumptions are needed due to the use of ordinal distances, which allow for direct comparison between the disparate data types. Using simulations, we test the performance of the method in identifying local transmission of disease in large datasets, and assess how sensitivity and specificity change with varying size of local transmission chains and varying overall disease incidence.

## Introduction

An essential question in contagious disease surveillance settings is whether cases result from local transmission within a population or from importation of infection from outside the population. This distinction is of importance, as interrupting local transmission calls for different interventions than stopping importation of infection. Unfortunately, distinguishing cases related through local transmission from cases that result from importation of infection is difficult.

When occurrences of a contagious disease are monitored and stored in a standardized way, statistical algorithms can aid in identification of local spread of the disease. For example, drug-resistant pathogens found in hospitals either are due to nosocomial transmission or are brought into the hospital by the patient. The former can be identified using surveillance data by assessing the number of cases in a fixed time period [1]; this identification is essential in optimizing hospital control measures.

Genetic sequence data of pathogens provide an informative data source for distinguishing between local transmission and importation. Sampled pathogens are now routinely genotyped or sequenced in many settings, offering the potential to distinguish cases that were infected in a local transmission chain from those that were infected elsewhere by evaluating small genetic differences between sampled pathogens. However, existing algorithms to find clusters of related cases in large datasets focus only on temporal data [2]–[8] or on spatiotemporal data [9]–[11]. Although genetic data are already being used to distinguish between different strains of the same species [1], [12], the full potential offered by these data has so far not been utilized [13].

**Figure 1:**
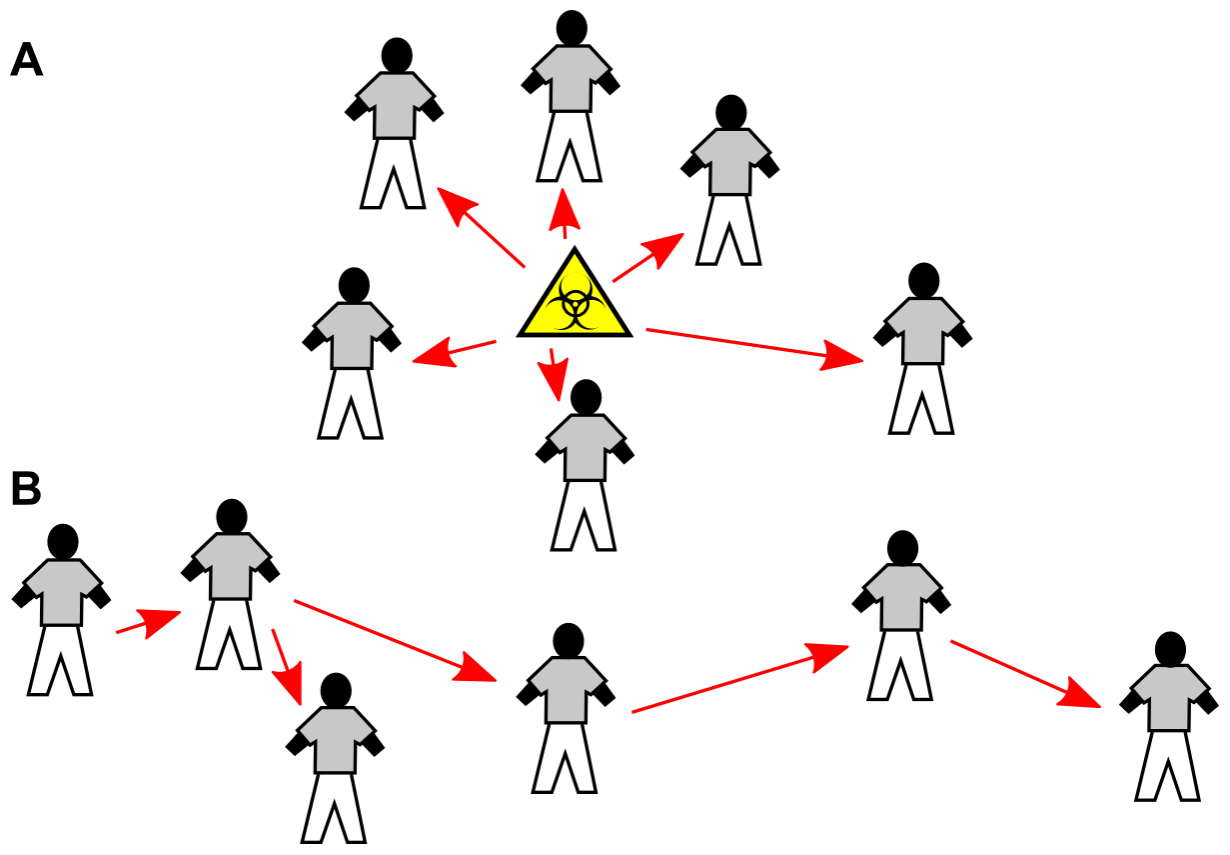
Different patterns of disease clusters. Clusters of disease cases caused by a point source (A) show a different pattern than clusters caused by human-to-human transmission of a contagious disease (B). (A) When there is a point source cases tend to be found in the region around it. Modern scan statistics exploiting this pattern have been developed to find evidence of point sources causing disease. (B) When contagious diseases are spread by human-to-human transmission, clusters tend to be more chain-like; the relevant distances are those between pairs of cases rather than between case and point source. Although it is still possible to find clusters in situation (B) with algorithms developed for (A), the problem can be handled more naturally by taking into account the different cluster pattern.

In outbreaks of contagious diseases, cases are caused by other cases. This property results in clusters of cases due to local transmission of contagious diseases having a different mathematical structure in space and time than clusters of cases due to non-contagious diseases. Clusters due to contagious disease tend to have a more chain-like shape (figure 1). However, existing clustering algorithms that focus on spatiotemporal data do not account for this property, as they often have not been developed specifically for contagious diseases.

Here, we present a method that identifies locally infected cases from a dataset containing genetic, temporal and geographical data. For each pair of cases, we assess the distance between these cases with respect to their locations, their times of symptom onset, and the genetic sequences of the pathogens isolated from the cases. For a pair of cases not related through local transmission, we expect the distances in the separate data types to be independent. For cases that are part of a local transmission chain, we expect the distances between these cases to be small for each of the separate data types. We employ a form of hierarchical clustering that uses an ordinal distance between cases based on their genetic, temporal and geographical distances, and that reflects the fact that for contagious diseases, cases are caused by other cases. Clusters of cases resulting from local transmission are identified by testing whether they have smaller pairwise distances than would be expected under a null hypothesis of independence between the location, time of symptoms onset, and pathogen sequence of cases. As the purpose of the present paper is to introduce and explain the methodology, and to illustrate its use and limitations, we use simulated datasets. To test the ability of the method to detect local transmission of a contagious disease, by assessing the sensitivity and false positive rate of assigning cases to local transmission clusters.

## Methods

We consider a contagious disease surveillance dataset that consists of a large number of cases. We assume that for each case we know the date of symptom onset or sampling date, the geographical location, and the genetic type or sequence of the pathogen. Some of the cases might be infected within the time and region of the study, while others are infected elsewhere. Our objective will be to identify transmission clusters; sets of cases related through a local transmission chain.

**Figure 2:**
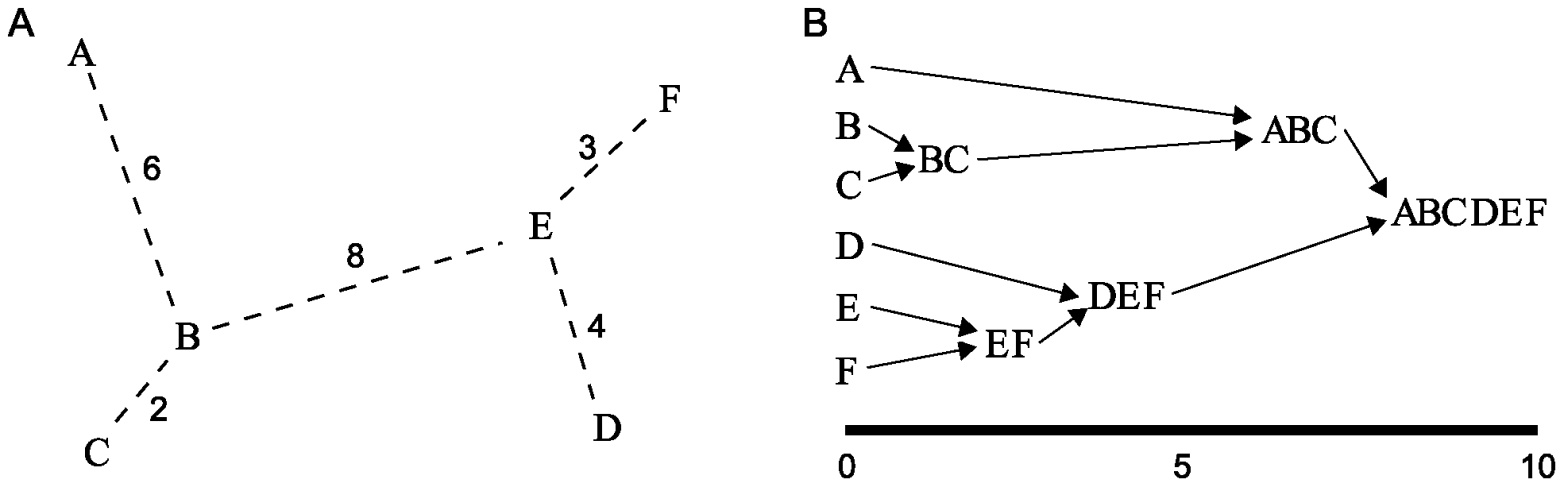
Graphical representation of hierarchical clustering. Hierarchical clustering sequentially clusters together elements of a set, based on inter-element distances. (A) Representation of a set of six elements. Shown is a minimal spanning tree: the tree that connects all elements minimizing total distance. (B) The clustering provided by hierarchical clustering when using single linkage clustering. Sequentially, the two current subsets with smallest distance are joined together, where the initial subsets are the six elements. This means the distances of clustering on the x-axis in (B) are the distances of the minimal spanning tree in (A). In total five distinct clusters are passed before all elements cluster together.

It is infeasible to consider every possible subset of cases in the dataset as a possible transmission cluster, because the number of subsets grows exponentially with the number of cases. We adopt a hierarchical clustering approach; here the dataset is sequentially divided into subsets of increasing size, yielding a tree-like structure, or dendogram (figure 2). The subsets encountered in this way are the most plausible local transmission clusters.

To perform hierarchical clustering one needs a measure of dissimilarity between sets of cases. We construct such a measure using both a measure of dissimilarity between individual cases and a linkage criterion that gives the similarity of two subsets as a function of the pairwise dissimilarities of their elements.

### Linkage criterion

We use single linkage clustering [14]–[16], the oldest and arguably simplest linkage criterion, which states that the distance between two sets of cases is the minimum of the pairwise inter-element distances. A commonly cited drawback of this criterion is that it tends to create chain-like clusters, with a high average distance within the cluster. Contagious disease epidemiology seems one of the few settings where this property is actually an advantage. Since contagious disease cases are caused by other cases the distance between two sets is well described by be the smallest pairwise distance and chain-like clusters are very plausible; for instance outbreaks spreading to another location, or viruses mutating in a certain direction over time.

### Pairwise dissimilarity

**Figure 3:**
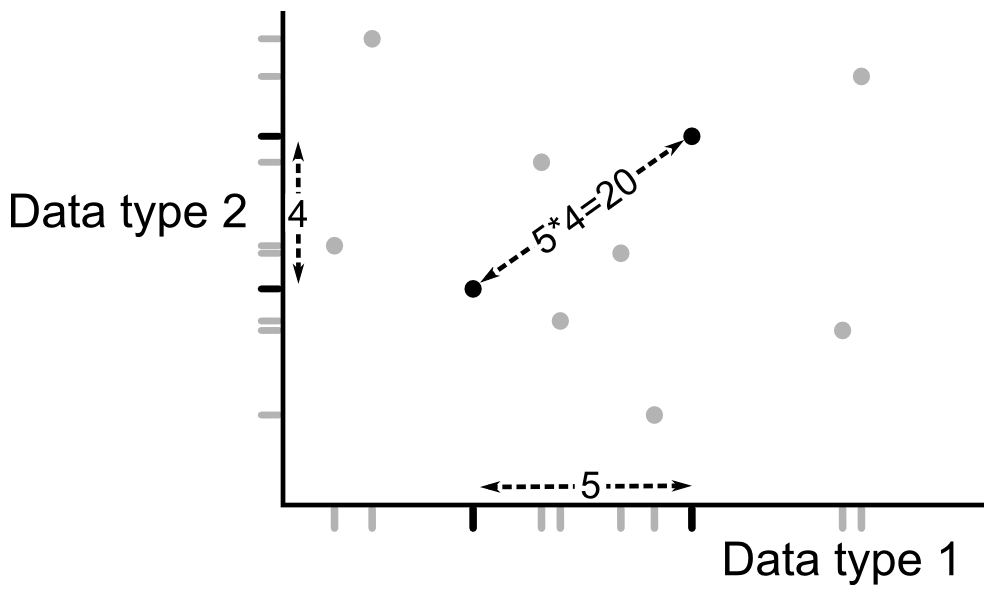
Graphical representation of the dissimilarity measure between cases. Shown is a dataset of nine cases and two (one-dimensional) data types. For each of the two data types, the dissimilarity between the two black cases is given as the number of cases in between them (for that data type), including one of the two black cases. This definition ensures the dissimilarity between a case and itself is zero. The total dissimilarity between the black cases is then given as the product of these, here 5*4 = 20.

It is not immediately obvious how a dissimilarity should be defined between two individual cases. We have to combine a temporal, a geographical and a genetic distance, comparing days with kilometers and mutations. Furthermore, the absolute values of these distances are not directly informative. First, because we assume no knowledge on pathogen characteristics we cannot interpret any absolute value. Second, because many cases in one geographical, temporal or genetic region might be the result of a high population density, seasonality or higher pathogen fitness, respectively, rather than of local transmission. The relevant notion of dissimilarity between two cases is therefore not an absolute distance, but the number of other cases found in between the two cases [17], i.e. closer to both the two cases than the two cases are to each other. For example, two cases living a kilometer apart are more likely to be related when this is in a rural area than in a large city, two cases infected at the same day are more likely to be related when they are infected during an off-season than during an epidemic, and two identical strains are more likely to be related when this is a rare sequence than when this sequence is ubiquitous. This relevant notion of number of cases in between two cases is not pathogen specific and allows for combination of the three disparate data types. We define the dissimilarity *d_i_* for a given data type *i* between two cases *a* and *b* as (figure 3):
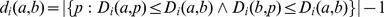
(1)where |.| denotes the number of elements of a set, ∧ the logical AND operator, *D_i_* the absolute distance for data type *i* (time, location or genetic) and the ‘−1’ ensures *d_i_*(*a,a*) = 0.

Under our null hypothesis of all cases being unrelated, the dissimilarities in the different data types are independent. In contrast, dissimilarities between two cases infected in the same local transmission chain will be small for each of the data types. We obtain the full dissimilarity *d* between two points *a* and *b* as the expected number of cases in between them under the null hypothesis; the product of the data type specific dissimilarities (figure 3)
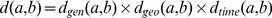
(2)When the data are continuous (i.e. all observed values are unique) it is possible to analytically obtain the full distribution for *d* under the null hypothesis. For instance, *d_time_* is distributed as the absolute value of the difference of two independent random variables following a discrete uniform distribution on [1,*N*], with *N* the number of cases. When cases are infected locally there will be more small dissimilarities than under the null hypothesis. When a data type is discrete (as genetic data always are) several cases can have identical values for this data type. In this case we propose an extension to *d_i_*(*a,b*) in which the dissimilarity between two cases is the expected number of cases in between them if this identical value was due to small measurement error (Appendix S1).

### Finding putative transmission clusters

We want to assess for a given subset *S* of the dataset *D* whether it is a local transmission cluster, i.e. whether the cases in *S* are closer together than would be expected under the null hypothesis. Define the unique ‘weakest link’ *l(S)* of a cluster as the largest dissimilarity in the minimal spanning tree of *S*; the larger the dissimilarity the less likely it is that all cases in the cluster are part of a local transmission chain. *l(S)* increases with *S*; we therefore compare *l(S)* to the value we would expect under the null hypothesis for a cluster of at least this size. We call *S* a putative transmission cluster (PTC) if the probability of observing a cluster of at least this size with weakest link at most *l(S)* under the null hypothesis is less than 0.001. This probability can be obtained by permuting the dataset (see Appendix S1 for details). The upper bound for the probability (here 0.001) should be small but other than that is arbitrary, and could be changed depending on the application.

It is important to note that the tests applied to each of the clusters encountered in the hierarchical clustering scheme are not independent. For example, if a set of ten cases is found to be a PTC then the set of eleven cases constructed by adding one random nearby case will probably also be a PTC. This dependence is inherent to clustering algorithms and not necessarily a problem, as finding the cluster is usually more important than uniquely identifying all the cases that belong to it. It could, however, lead to a high false positive rate when assessing whether cases are correctly assigned to a PTC.

### Testing on simulated datasets

To gauge the strengths and limitations of the algorithm, we tested it on simulated datasets where we know precisely which cases were part of a local transmission chain and which were import. The performance of any clustering algorithm depends on how strongly the clusters are separated in the data. For instance, if a dataset consists of several outbreaks clearly distinguishable in time and place, we expect an algorithm to do well. On the other hand, when cases belonging to one outbreak can be found throughout the spatiotemporal and genetic space, any algorithm will struggle to identify the outbreak. The simulations are thus focused on the intermediate region, where clustered cases are not easily distinguishable based on separate data types, but are still close enough that the combined information from the data types yields enough information for clustering.

We use two measures of the performance of the proposed method. First, we take the percentage of locally infected cases correctly assigned to a PTC. Second, we take the percentage of imported cases incorrectly assigned to a PTC. The former is a measure of the sensitivity, the latter of the false positive rate. If sensitivity is high whilst the false positive rate remains low, the method could be suitable for use in outbreak detection. As we assess performance of the method at the case level, while statistical tests are performed at the cluster level, the false positive rate is not guaranteed to be beneath the p-value of 0.001 used. If the false positive rate becomes too large the method would be unsuitable for outbreak detection, as too many false alarms would be given, but might still be useful in assessing properties of locally infected cases. We performed simulations under different incidence rates and with different sizes of the transmission clusters.

Each of the simulated datasets consisted of many unrelated cases and a small number of local transmission clusters, containing in expectation ten percent of the total number of cases for each of the simulations. All of the cases have a time, position and genotype associated with them. The geographical position of an imported case *A* is given by *x*_A_∼(Uniform(0,100), Uniform(0,100)), its time of sampling by *t_A_*∼Uniform(0,100), and its genotype *gen_A_* is represented by a string of 8 random bits (each can be 0 or 1 with equal probability). Locally infected cases were simulated with infectors chosen as specified below. An infected case *B* and its infecting case *A* are related as follows; *x_B_∼x_A_*+(Normal(0,4), Normal(0,4)), *t_B_∼t_A_*+Exponential(1), and *gen_B_* is generated from *gen_A_* by flipping each bit with probability 1/16.

The absolute spatial distance between two cases was taken to be the usual Euclidean distance, with distances in both dimensions the minimum of *|x_1_−x_2_|* and 100−*|x_1_−x_2_|*. This makes the geographical region a torus, ensuring all clusters are fully observed. The absolute genetic distance was calculated as the number of different bits, leading to an expected genetic distance of 0.5 between infector and infected. The absolute temporal distance was the absolute value of the time difference. From these absolute distances dissimilarities were calculated for each data type, and combined into pairwise dissimilarities using equation (2).

In a first scenario all locally infected cases belong to the same outbreak, with an index case randomly chosen from the unrelated cases. In a second scenario we generated smaller transmission clusters, by letting 1/9 of all cases generate secondary cases according to a geometric distribution with mean R = 0.5. These were themselves equally infectious, yielding transmission clusters of expected size 2. In the epidemiological literature this scenario is known as ‘stuttering transmission chains’ [18]; small outbreaks occur but large outbreaks do not, since the mean number of secondary cases per infectious case, or effective reproduction number, R, is smaller than one. In a third scenario we generated even more and smaller outbreaks, with each case generating new cases according to a geometric distribution with mean R = 0.1, yielding a dataset with many very small transmission clusters of expected size 1.11. For all of these scenarios, we performed simulations with an initial number of unrelated cases of 90, 450 or 900, representing different incidence rates, yielding nine simulation scenarios in total. The expected total number of cases for these simulations are thus 100, 500 and 1000. For each of these nine scenarios, we simulated 100 datasets, and applied the methodology to identify significant clusters.

The results of the clustering algorithm depend on how related infector-infected pairs are in each of the data types. When this relation is stronger, we expect clustering results to improve. We therefore performed additional simulations where the distances between infector-infected pairs are smaller (Appendix S1).

Many actual surveillance datasets face the problem of missing or unobserved cases. This is similar to a scenario where all cases are observed, but the relation between infector-infected pairs is weakened. To illustrate this, we performed analyses on simulated datasets from which we randomly discarded 20% of all cases (Appendix S1).

## Results

**Figure 4:**
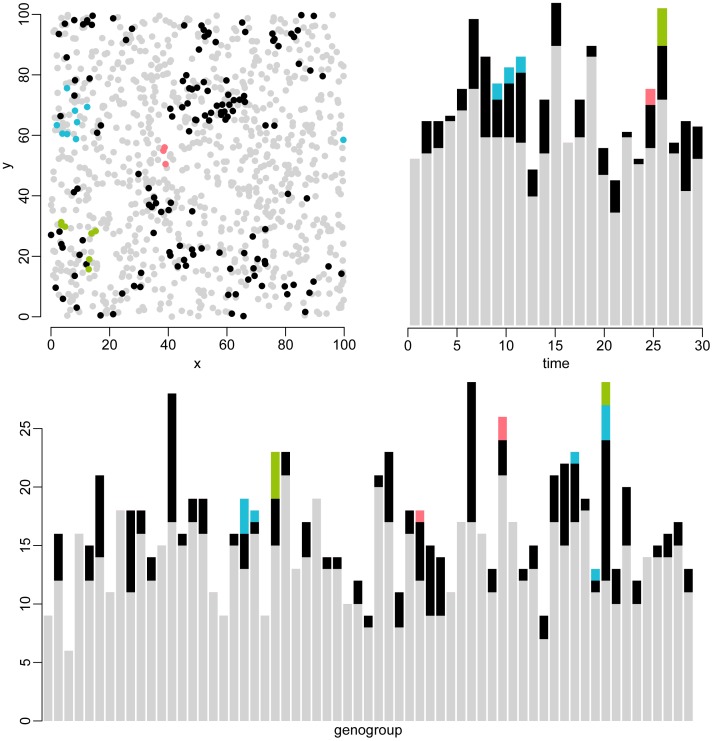
Graphical representation of the three data types for a typical simulation. This simulation consisted of 1019 cases of which 119 (12%) were infected by other cases. In total, there were 158 related cases belonging to 39 transmission chains, and 861 unrelated cases (gray). To visualize individual transmission chains, three chains were chosen at random and drawn in blue, green and pink. (A) Geographical location of all simulated cases. The geography is a torus, so the right side is equated with the left side, and the top side is equated with the bottom side. (B) Simulated cases over time. (C) Simulated cases have one of 2^8^ = 256 possible genotypes. For clarity, the distribution of cases over 64 genogroups is plotted; a genogroup is defined as a set of four genotypes that are identical up to the last two digits. The order of the genogroups on the x-axis does not reflect genetic distance. Note that outbreaks cannot be accurately identified using only one of these data types.

Figure 4 gives a graphical representation of a typical simulated dataset with small outbreaks for each of the separate data types. Figure S1 gives the same information for a simulated large outbreak (see Supporting Information for corresponding simulated datasets). Under the chosen parameter settings, identifying the different clusters by only looking at the separate data types is challenging.

**Figure 5:**
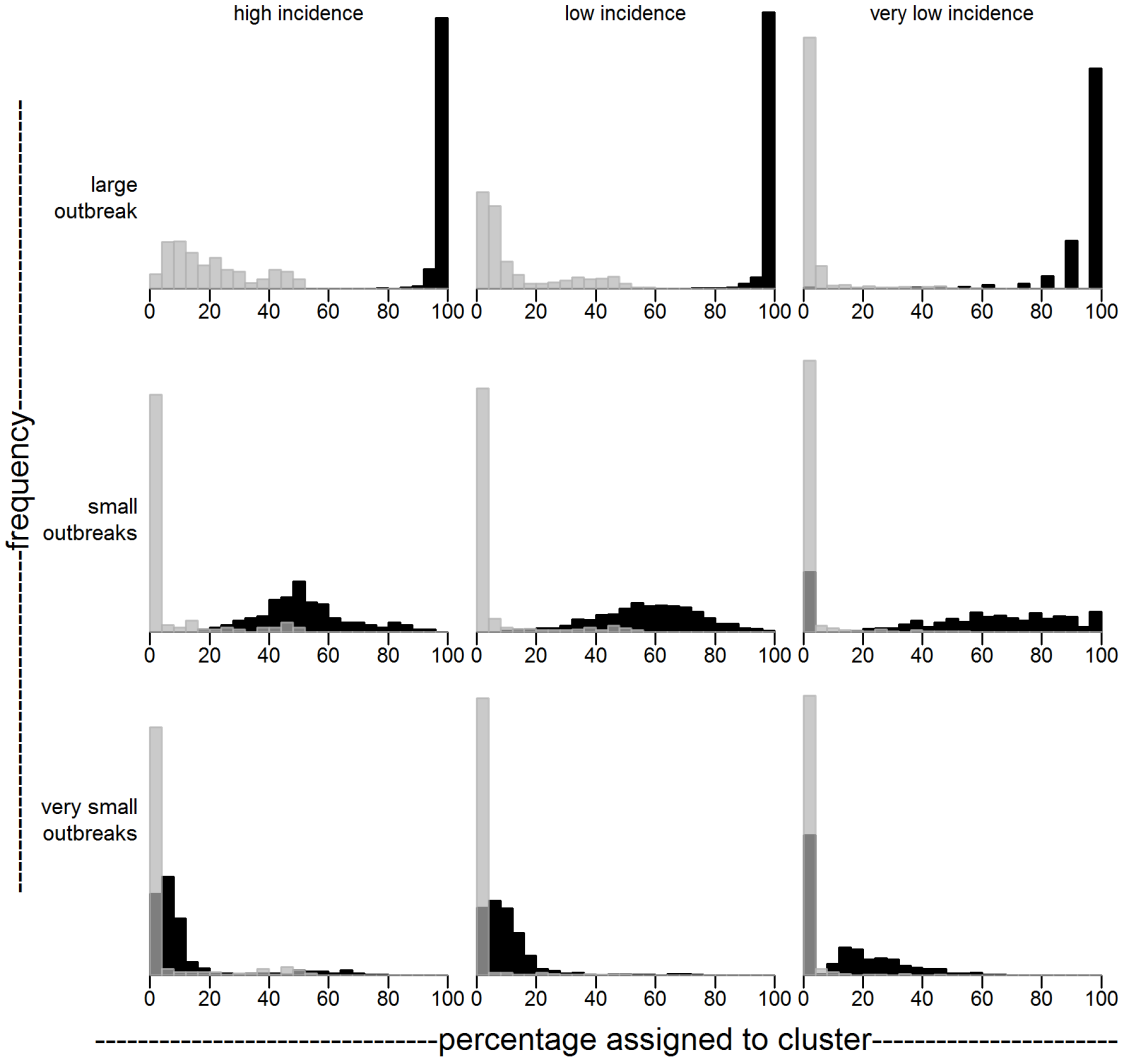
Sensitivity (black) and false positive rate (gray) for analyses on simulated datasets. For each of nine simulation scenarios, percentage of outbreak (black) and non-outbreak (gray) cases assigned to a putative transmission cluster are shown. In each scenario, ten percent of all cases are an outbreak case. Total expected number of cases is (left column) 1000, (middle column) 500 or (right column) 100. Outbreak cases belong to (top row) one large outbreak, (middle row) small outbreaks caused by 1/10 of cases being contagious with basic reproduction number R = 0.5, (bottom row) very small outbreaks caused by all cases being contagious with R = 0.1. For all scenarios, outbreak cases are distinguishable from unrelated cases. Sensitivity increases with outbreak size, at the cost of an increased false positive rate. Sensitivity and false positive rate improve when the incidence, or equivalently the number of cases in the same region of spacetime, decreases. Figure 4 corresponds to a simulation from the middle left panel.

The results for finding local transmission for all simulation scenarios are given in figure 5 and table 1. For each simulation scenario, we report the distribution and median of the percentage of cases assigned to a putative transmission cluster (PTC), both for locally infected and for imported cases. For all scenarios, the percentage of locally infected assigned to a PTC is higher than the percentage of import cases assigned to a PTC. This means that for all scenarios the three data types, when combined, provide sufficient statistical signal to identify local transmission.

**Table 1:** Median of sensitivity/false positive rate of assigning locally infected cases to a putative transmission cluster for simulated datasets.

	High incidence	Low incidence	Very low incidence
Large outbreak	1.00/0.16	1.00/0.06	1.00/0.01
Small outbreaks	0.49/0.01	0.58/0.01	0.60/0.00
Very small outbreaks	0.06/0.00	0.08/0.00	0.11/0.00

In general, the method assigns outbreak cases to PTC's more often when transmission clusters are larger. This is no surprise as the strength of the statistical signal increases with outbreak size. This higher sensitivity comes at the cost of a lower specificity; when assessing the large outbreaks of expected size 100 the median false positive rate is 0.16, while it is near 0 for most other scenarios. The false positives here are cases that are, coincidentally, close to the actual cluster; the number of such cases increases with the size of the outbreak.

The method has a lower sensitivity and specificity when the incidence rate is higher. This is because there are more unrelated cases per unit of space, time and genetics, while the absolute inter-outbreak case distances remain the same. Therefore, the ordinal distance between outbreak cases becomes larger when incidence rates are higher, which makes it harder to identify transmission clusters (figure S3, table S2). When inter-outbreak case distances become smaller, outbreaks are easier to detect (see Appendix S1, figure S2, table S1).

## Discussion

We have presented a method to identify transmission chains of locally infected contagious disease cases in large databases containing temporal, geographical and genetic data. The method does not require assumptions on population at risk or pathogen-specific properties. The method is novel in explicitly incorporating the genetic distances measured between sampled pathogens, and in accounting for the chain-like structure of transmission chains of contagious diseases.

Several methods to find locally infected cases in large datasets have been published and some are commonly used in epidemiological investigations [1], [9], [12], [13]. However, many of these methods were not explicitly developed for analysis of contagious diseases, and ignore the fundamental characteristic of contagious diseases: that each infected case can itself be a source for new cases. The method presented here does take this into account by focusing on the distances between cases, rather than on the number of cases in a particular region of space-time. This latter approach is suitable when cases are caused by one common source, rather than by the cases themselves.

The ability of the method to correctly cluster together cases belonging to the same transmission chain depends on how ‘close’ these cases are to each other in time, space and genetics, relative to non-related cases. This depends both on the properties of the pathogen studied, the incidence of disease, and the size of the study region and duration. For example, a dataset resulting from a study period of one year on a pathogen with an average serial interval of half a week would for our method, due to the ordinal distances used, be equivalent to a dataset resulting from a study period of ten years on a pathogen with a serial interval of five weeks and ten times lower incidence. Shorter serial intervals, lower incidence rates and longer study periods allow for more accurate identification of outbreak cases.

In our simulations, we have taken the Euclidean distance between geographical locations. However, this is not always the most relevant distance metric. For example, people are more likely to travel between densely populated areas than between sparsely populated areas [19]. Thus, when a study region encompasses both urban and rural areas, more relevant measures of distance could be given by mobility patterns [20] or road distances [21]. Note that no extra correction is needed to adjust for the higher urban population densities leading to more cases; this is taken into account by the ordinal distance used.

The non-parametric method introduced is able to identify locally infected cases when little is known about the pathogen studied. When precise pathogen-specific information or information on population at risk is available, a more precise description of the system can be given. More specific methods can be used and information can be obtained from the data types separately, which should lead to better identification of transmission clusters. The non-parametric method could still be of value in such as a scenario, as it provides a simple first-try approach: results can be compared to those obtained from an analysis that uses more information, and assumptions made about pathogen characteristics and population at risk can be tested.

Here we performed validation of the method using simulated datasets, in which the origin of cases is known. Results obtained give confidence the method can be sensibly applied to actual surveillance datasets. Examples of existing large molecular epidemiological databases include VNTR typing datasets of tuberculosis [22], spa typing datasets of MRSA [23] and short read sequencing datasets of hepatitis B, hepatitis A and norovirus [24]–[26]. Note that the relevant spatial information differs for these datasets; we might focus on place of residence for tuberculosis, but on hospital, ward or even bed for MRSA. Future work will have to focus on applying the method presented here to such datasets.

The method presented has some drawbacks. First, the null hypothesis states that all cases in the dataset are independent. This leads to a bias when many transmission clusters are present; as the locally infected cases cluster together, the remaining independent cases will themselves lie closer together (in ordinal distance) than under the null hypothesis. Thus, especially when a high percentage of cases are locally infected, the statistical test could overestimate the percentage of clustered cases. This overestimation might be alleviated if prior information on the percentage of cases locally infected were available. Second, we assumed independence between the different data types for unrelated cases. These data are never truly independent, as all cases belong to the same phylogenetic tree acting over a long time scale. The local outbreaks we are interested in can be seen as local tips of these large trees. Whether the data can be approximated as being independent depends on the spatial dynamics of the pathogen, the evolutionary time separating sampled pathogens and the size of the region studied. For example, the approximation might be valid when studying MRSA at the hospital level, but not at the level of a continent as geographical structure can be seen in genotypes sampled [23]. Third, we have not taken into account the boundaries of our datasets, i.e. the edges of the geographical area and time window studied. This might decrease the sensitivity of finding clusters near the start or end of the study period, and should be addressed when the method is applied prospectively.

Results of clustering methods such as the one presented are important in epidemiological investigations for a number of reasons. First, they provide a measure of how much local transmission takes place. For example, if many putative transmission clusters are found in a hospital setting, infection control measures have to be intensified. Second, the algorithm can be used as a tool to find transmission clusters in large databases that can then be further investigated, removing the need for a detailed analysis by hand of the complete database. Third, properties of clustered cases can be compared to non-clustered cases. This will, for example, allow researchers to test whether patients of a particular age are more prone to transmit disease, or whether certain genotypes are more likely to spread in a hospital setting. These applications differ in their requirements on the sensitivity and specificity of the algorithm, where generally the second application will have the most stringent, and the third application the most relaxed requirements.

With the decreasing cost of sequencing and genotyping techniques, the availability of genetic data continues to grow. In particular, in many surveillance settings large molecular epidemiological databases have been set up. As the size and complexity of these databases grows, we can only expect the usefulness of automated methods such as these to assist in answering public health questions will grow concordantly.

## Supporting Information

Appendix S1 **Details on calculations and simulations, and additional simulation scenario results.**
(PDF)

Figure S1 **Graphical representation of the three data types for a typical simulation containing one large outbreak.** This simulation consisted of 1000 cases of which 10% pertained to one large outbreak (black). (top left) Geographical location of all simulated cases. The geography is a torus, so the right side is equated with the left side, and the top side is equated with the bottom side. (top right) Simulated cases over time. (bottom) Simulated cases have one of 28 = 256 possible genotypes. For clarity, the distribution of cases over 64 genogroups is plotted; a genogroup is defined as a set of four genotypes that are identical up to the last two digits. The order of the genogroups on the x-axis does not reflect genetic distance.
(PDF)

Figure S2 **Sensitivity and false positive rate when distances between pairs of infector and infected are small.** Percentage of (black) outbreak and (gray) non-outbreak cases assigned to putative transmission clusters for simulations under nine different scenarios, when the distance between a locally infected case and its infector is smaller than in the simulations given in the main text. In each scenario, 10% of all cases is an outbreak case. Total expected number of cases is (left column) 1000, (middle column) 500 or (right column) 100. Outbreak cases belong to (top row) one large outbreak, (middle row) small outbreaks caused by 1/10 of cases being infectious with R = 0.5, (bottom row) minor outbreaks caused by all cases being infectious with R = 0.1. Sensitivity and specificity increase with respect to simulations in the main text, as smaller distances lead to a stronger statistical signal.
(PDF)

Figure S3 **Sensitivity and false positive rate when 20% of cases are unobserved.** Percentage of (black) outbreak and (gray) non-outbreak cases assigned to putative transmission clusters for simulations under nine different scenarios, when 20% of cases is unobserved. In each scenario, 10% of all cases is an outbreak case. Total expected number of cases is (left column) 1000, (middle column) 500 or (right column) 100. Outbreak cases belong to (top row) one large outbreak, (middle row) small outbreaks caused by 1/10 of cases being infectious with R = 0.5, (bottom row) minor outbreaks caused by all cases being infectious with R = 0.1. As expected, performance decreases when the distance between cases increases. A notable exception are the very small clusters, where sensitivity actually increases. As these transmission clusters are mainly of size two, discarding a case does not lead to larger distances, but to elimination of the cluster. Thus the number of cases and clusters is affected, but the intra-cluster distances are not.
(PDF)

Table S1 **Median of sensitivity/false positive rate of assigning locally infected cases to a putative transmission cluster for simulated datasets where distances for infector-infected pairs are small.**
(DOCX)

Table S2 **Median of sensitivity/false positive rate of assigning locally infected cases to a putative transmission cluster for simulated datasets where 20% of cases are unobserved.**
(DOCX)

Simulated Dataset S1 **Example simulated dataset, used to generate** **figure 4** **.**
(TXT)

Simulated Dataset S2 **Example simulated dataset, used to generate figure S1.**
(TXT)
