# Supplementary material for: Finding Evidence for Local Transmission of Contagious Disease in Molecular Epidemiological Datasets
Source: PLoS One. 2013 Jul 26;8(7):e69875. doi: 10.1371/journal.pone.0069875 (PMC3724731; doi:10.1371/journal.pone.0069875)
Supplement: Appendix S1 — Details on calculations and simulations, and additional simulation scenario results. (PDF) [file pone.0069875.s001.pdf]

Appendix

Finding evidence for local transmission of contagious disease in molecular epidemiological datasets

Rolf J.F. Ypma, Tjibbe Donker, W. Marijn van Ballegooijen, Jacco Wallinga

Contents

1 Pairwise dissimilarities 2

2 Putative transmission clusters 2

3 Details of simulations 4
- 3.1 Generating simulated datasets 4
- 3.2 Final size calculations 4

4 Additional simulation results 5
- 4.1 Small distances 5
- 4.2 Unobserved cases 5

1 Pairwise dissimilarities

Let $D_i^m(a,b)$ be the measured distance between two cases a and b for data type i. There could be identical values in our dataset (i.e. $D_i^m(a,b) = 0$). As we use ordinal distances to detect cases lying close together, detection of local transmission clusters will be more challenging when many values are identical; no ordering exists on these. To be able to make comparison between cases with identical values and those with distinct values, we will assume that for all cases for which the same value was measured, the actual value lies a random infinitesimal distance away from this measured value. This is actually true for the temporal data, which is always interval censored, as dates but not exact times are given. It is not true for genetic data, which are discrete. However, these can be seen as a proxy for evolutionary time separating two samples, which is again continuous.

We define the dissimilarity $d_i(a,b)$ between two cases a and b as the expected number of cases between them, plus one:

$$\begin{aligned} d_i(a,b) &= |\{p : D_i^m(a,p) < D_i^m(a,b) \wedge D_i^m(b,p) < D_i^m(b,a)\}| + \tfrac{|\{p:D_i^m(a,p)=0\}-1|+|\{p:D_i^m(b,p)=0\}-1|}{2} + 1 \\ &= |\{p : D_i^m(a,p) < D_i^m(a,b) \wedge D_i^m(b,p) < D_i^m(b,a)\}| + \tfrac{|\{p:D_i^m(a,p)=0\}|+|\{p:D_i^m(b,p)=0\}|}{2} \end{aligned}$$

when $D_i^m(a,b) \neq 0$, and

$$d_i(a,b) = \frac{|\{p : D_i^m(a,p) = 0\}| - 2}{3} + 1 = \frac{|\{p : D_i^m(a,p) = 0\}| + 1}{3}$$

when $D_i^m(a,b) = 0$. Here '$\wedge$' denotes the logical AND operator; $A \wedge B$ is true if and only if both A and B are true. To see that the definition above coincides with the expected value plus one, consider three points a, b and c, with a and b having the same observed value ()$D_i^m(a,b) = 0$), each lying a random infinitesimal distance away from their measured values. Then the probability that any two of the actual pairwise distances are equal is zero. Therefore, if $D_i^m(a,c) \neq 0$, the probability that b is in between a and c is $\frac{1}{2}$. If $D_i^m(a,c) = 0$, the probability that b is in between a and c is $\frac{1}{3}$. Further note that, for distinct cases, when identical values do not occur in our dataset the definition above is equivalent to equation (1) in the main text.

As in the main text, the full dissimilarity between two cases a and b is given by

$$d(a,b) = \Pi_i d_i(a,b)$$

2 Putative transmission clusters

For any subset $S \subseteq D$, define $l(S)$ as the largest dissimilarity in the minimum spanning tree of S. Note that several minimum spanning trees can exist, but $l(S)$ is unique (see lemma 1). To test the null hypothesis of independence between data types, we construct the set D' from D by randomly permuting the values of the data types. D' is identical to D for each of the data types, but satisfies the null hypothesis. We then define the

p-value for S as the probability that a subset with at least that size and at most that largest dissimilarity exists under the null hypothesis:

$$P(\exists S' \subseteq D' : |S'| \geq |S|, l(S') \leq l(S))$$

and we call S a putative transmission cluster (PTC) if this p-value is beneath a threshold of 0.001.

We can limit the number of clusters we have to test using hierarchical clustering, a technique that yields a dendogram of the dataset. A dendogram can be defined as a function $h : [0, \infty) \to P_D$, where P_D is the set of all partitions of the dataset D, with the properties that $m \leq m'$ implies $h(m) \leq h(m')$ (i.e. every element of $h(m)$ is a subset of an element of $h(m')$), and h is eventually the whole dataset ($h(m) = D$ for sufficiently large m). $h(m)$ here is the set of subsets S of D such that $l(S) \leq m$ and the only set S_2 that contains S and has $l(S_2) \leq m$ is S itself. Let $\mathcal{S}$ be the set of subsets of D that are in $h(m)$ for some m. By lemma 2, subsets of D that are a PTC are always contained in an element of $\mathcal{S}$ which is also a PTC. Since we are interested in whether cases belong to a cluster or not, we only have to test the elements of $\mathcal{S}$ for being a PTC.

Lemma 1. *For any weighted graph G, all minimal spanning trees have the same maximum edge weight.*

To prove this, let's assume T_1 and T_2 are minimal spanning trees of G, such that their maximum edge weights are different. Without loss of generality, let the maximum edge weight of T_1 be larger, and let $e \in T_1$ be an edge with this weight. Now select an edge e' from T_2 such that e' is in the cut induced by e in T_1. As the maximum edge weight of T_2 is smaller than that of T_1 by assumption, the weight of e' is smaller than that of e. The tree $(T_1 - \{e\}) \cup e'$ is a spanning tree of G, with total weight less than T_1. This is a contradiction, as T_1 was a minimal spanning tree. □

Lemma 2. *If $S \subseteq D$ is a putative transmission cluster (PTC), $\exists T \in \mathcal{S}$ with $S \subseteq T$ and T a PTC.*

Either $S \in h(l(S)) \subseteq \mathcal{S}$ and we are done, or $\exists T \in h(l(S)) \subseteq \mathcal{S}$, with $S \subset T$. Because S is a PTC we have

$$P(\exists S' \subset D' : |S'| \geq |S|, l(S') \leq l(S)) < 0.001$$

since furthermore $|T| > |S|$, $l(T) = l(S)$ and l is monotonically increasing in cluster size, we have that

$$\begin{aligned} P(\exists S' \subset D' : |S'| \geq |T| > |S|, l(S') \leq l(T) = l(S)) &\quad <= \\ P(\exists S' \subset D' : |S'| \geq |S|, l(S') \leq l(S)) &\quad < \quad 0.001 \end{aligned}$$

which shows that T is also a PTC. □

3 Details of simulations

3.1 Generating simulated datasets

In our first simulation scenario, all locally infected cases belong to one large outbreak. One index case was generated near the start of the study period so the outbreak would be completed within the time window. As the variance in the final size of a large outbreak generated by branching processes is quite large, we restricted this outbreak to be of size exactly one tenth of the size of the total simulated dataset. We therefore generated cases until the number was reached, and picked an infector for each from the set of previously generated cases. As assigning cases randomly from the set of already generated cases would amount to strong superspreading behavior (the index case would get a large number of infectees assigned), we preferentially picked more recently generated cases. In particular, we set the probability for any generated case to be assigned as an infector as twice the probability of picking the case generated before. For example, when three cases had been generated, they would be picked as an infector with probabilities 1/7, 2/7 and 4/7. This procedure is arbitrary, but simple and keeps the expected number of infections per infected individual bounded. For example, the expected number of infections caused by the index case would be $\sum_{i=1}^{N} \frac{1}{2^i-1} \approx 1.61$.

The small and very small outbreaks were generated using branching processes, as explained in the main text. Below we calculate the expected size of the outbreaks generated in this way.

3.2 Final size calculations

To find the expected value of the final size S of the outbreaks in the second and third scenario, let $f(x)$ be the probability that one infectious case infects x others. For the geometric distribution we use, $f(x) = p^x(1-p)$, where $p = \frac{R}{1+R}$ and R is the expected number of infections per infectious case. As each case infected again infects new cases, we have

$$E(S) = 1 + \sum_{x=0}^{\infty} f(x)xE(S) = 1 + RE(S)$$

which simplifies to $E(S) = \frac{1}{1-R}$, yielding expected sizes 2 and $\frac{10}{9}$ for the R values of 0.5 and 0.1 used.

As only outbreaks of value of at least 2 are characterized as clusters in our analysis, we might also want to find the expected size of these clusters: $E(S|S > 1)$. This is equivalent to conditioning on the index case causing at least one infection. We get

$$E(S|S > 1) = 1 + \sum_{x=1}^{\infty} \frac{f(x)}{1-f(0)} xE(S) = 1 + \frac{R}{1-f(0)} E(S) = 1 + \frac{(1+R)}{1-R}$$

yielding 4 and 20/9≈2.22 for the two scenarios.

4 Additional simulation results

In this section we give the results obtained by applying the proposed method to simulations where the absolute distances between infector-infected pairs are smaller than in the main text, and to simulations where 20% of cases are unobserved.

4.1 Small distances

The statistical signal left by clusters of cases depends for a large part on the relation for each of the data types between infector-infected pairs. When distances in these data types are smaller, the statistical signal is stronger. To illustrate this, we performed additional simulations in which these distances are smaller. We simulate as described in the main text, but the time distance is now exponentially distributed with expectation 0.5 (1 in the main text), the geographical distance is $N(0, 2)$ ($N(0, 4)$ in the main text), and the expected number of mutations is now 0.1 (0.5 in the main text). Clustering performance is given in figure S2 and table S1. As the statistical signal is much stronger, the distinction between outbreak and unrelated cases is much clearer than in the main text.

4.2 Unobserved cases

Many datasets face the problem of missing or unobserved cases. Here, we tested the performance of our method when facing unobserved cases. We do this by performing simulations as described in the main text, and then separately discarding each of the cases with probability 0.2, thus discarding 20% of cases at random. We applied our method to this reduced datasets, results are given in figure S3 and table S2. Datasets with missing cases are similar to complete datasets with larger distances between the cases; thus this scenario constitutes the opposite of the one in the previous section. As expected, clustering performance decreases for most scenarios. A notable exception are the very small clusters, where sensitivity actually increases. As these transmission clusters are mainly of size two, discarding a case does not lead to larger distances, but to elimination of the cluster. Thus the number of cases and transmission clusters is affected, but not the intra-cluster distances. Outbreak cases and unrelated cases can be distinguished for all scenarios, showing that the method can provide useful results even when cases are unobserved.
