# Supplementary figures and images for: Finding Evidence for Local Transmission of Contagious Disease in Molecular Epidemiological Datasets
Source: PLoS One. 2013 Jul 26;8(7):e69875. doi: 10.1371/journal.pone.0069875 (PMC3724731; doi:10.1371/journal.pone.0069875)

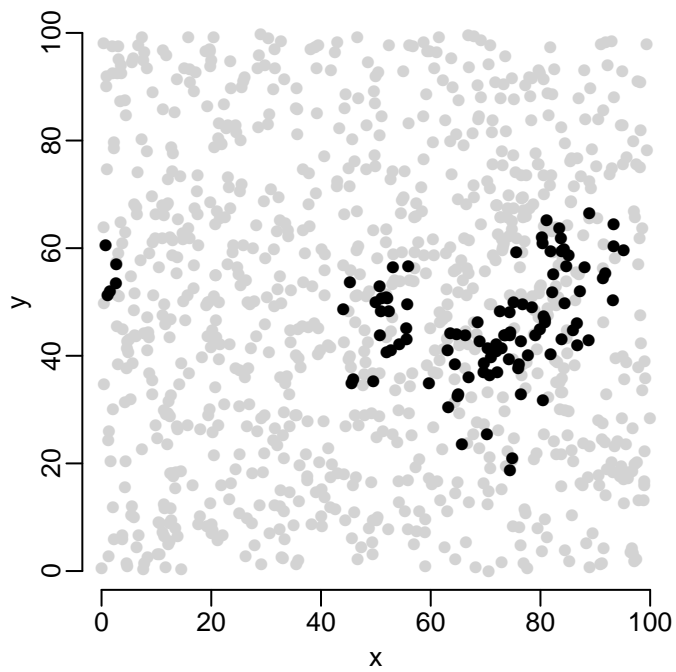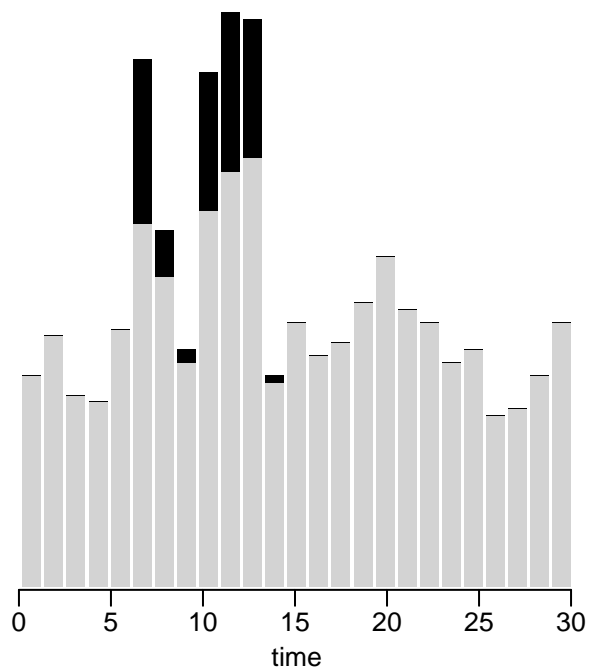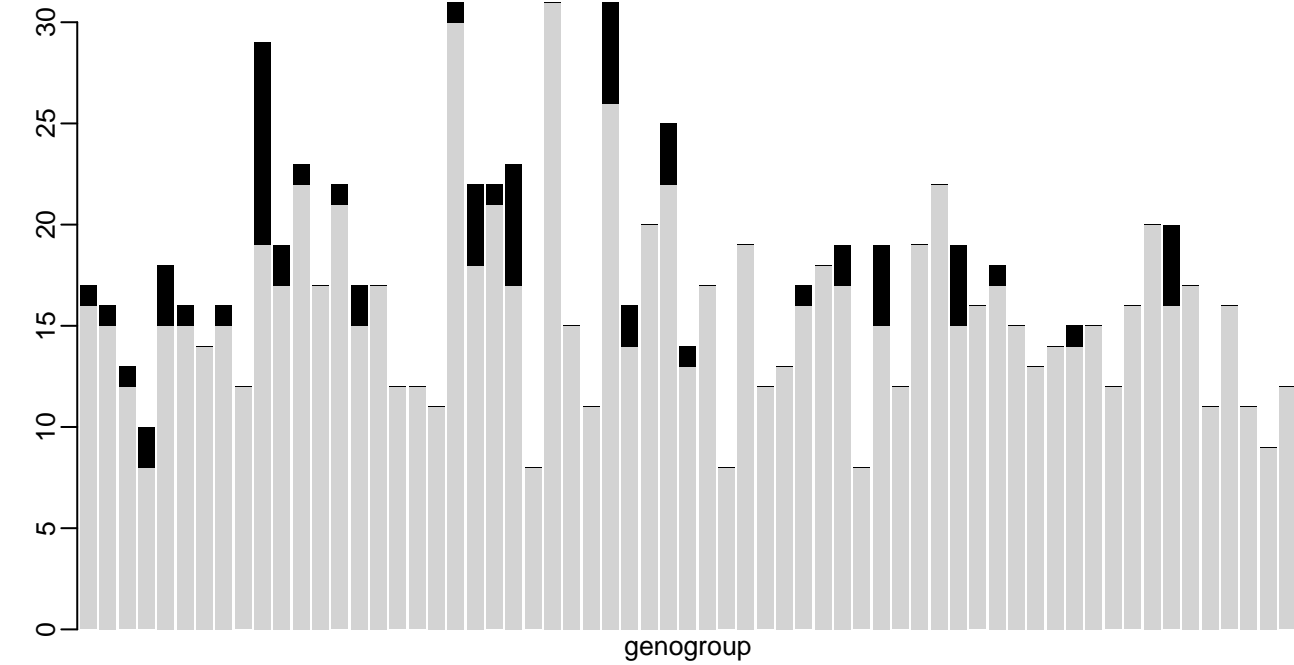

Supplement: Figure S1 — Graphical representation of the three data types for a typical simulation containing one large outbreak. This simulation consisted of 1000 cases of which 10% pertained to one large outbreak (black). (top left) Geographical location of all simulated cases. The geography is a torus, so the right side is equated with the left side, and the top side is equated with the bottom side. (top right) Simulated cases over time. (bottom) Simulated cases have one of 28 = 256 possible genotypes. For clarity, the distribution of cases over 64 genogroups is plotted; a genogroup is defined as a set of four genotypes that are identical up to the last two digits. The order of the genogroups on the x-axis does not reflect genetic distance. (PDF) [file pone.0069875.s002.pdf]

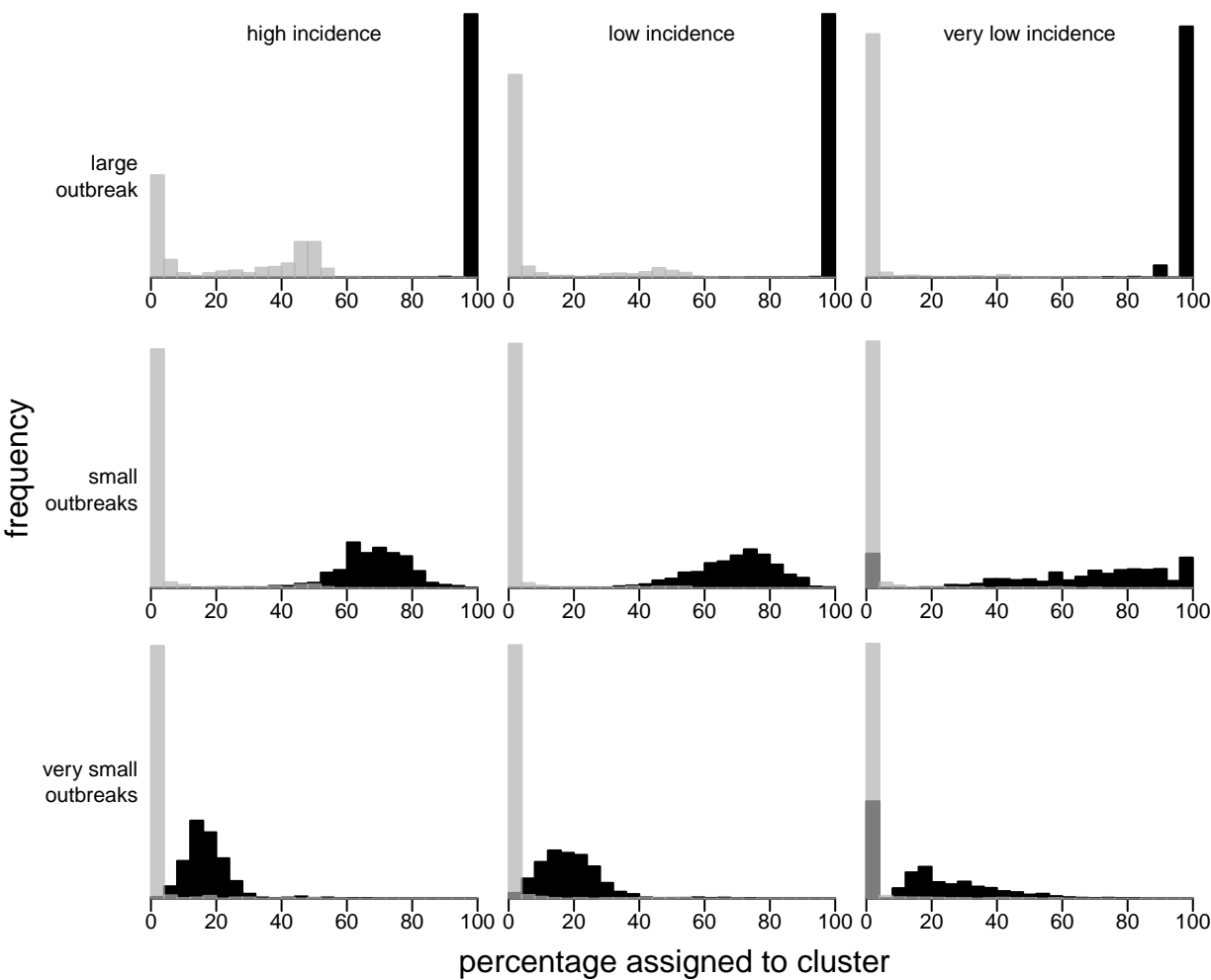

Supplement: Figure S2 — Sensitivity and false positive rate when distances between pairs of infector and infected are small. Percentage of (black) outbreak and (gray) non-outbreak cases assigned to putative transmission clusters for simulations under nine different scenarios, when the distance between a locally infected case and its infector is smaller than in the simulations given in the main text. In each scenario, 10% of all cases is an outbreak case. Total expected number of cases is (left column) 1000, (middle column) 500 or (right column) 100. Outbreak cases belong to (top row) one large outbreak, (middle row) small outbreaks caused by 1/10 of cases being infectious with R = 0.5, (bottom row) minor outbreaks caused by all cases being infectious with R = 0.1. Sensitivity and specificity increase with respect to simulations in the main text, as smaller distances lead to a stronger statistical signal. (PDF) [file pone.0069875.s003.pdf]

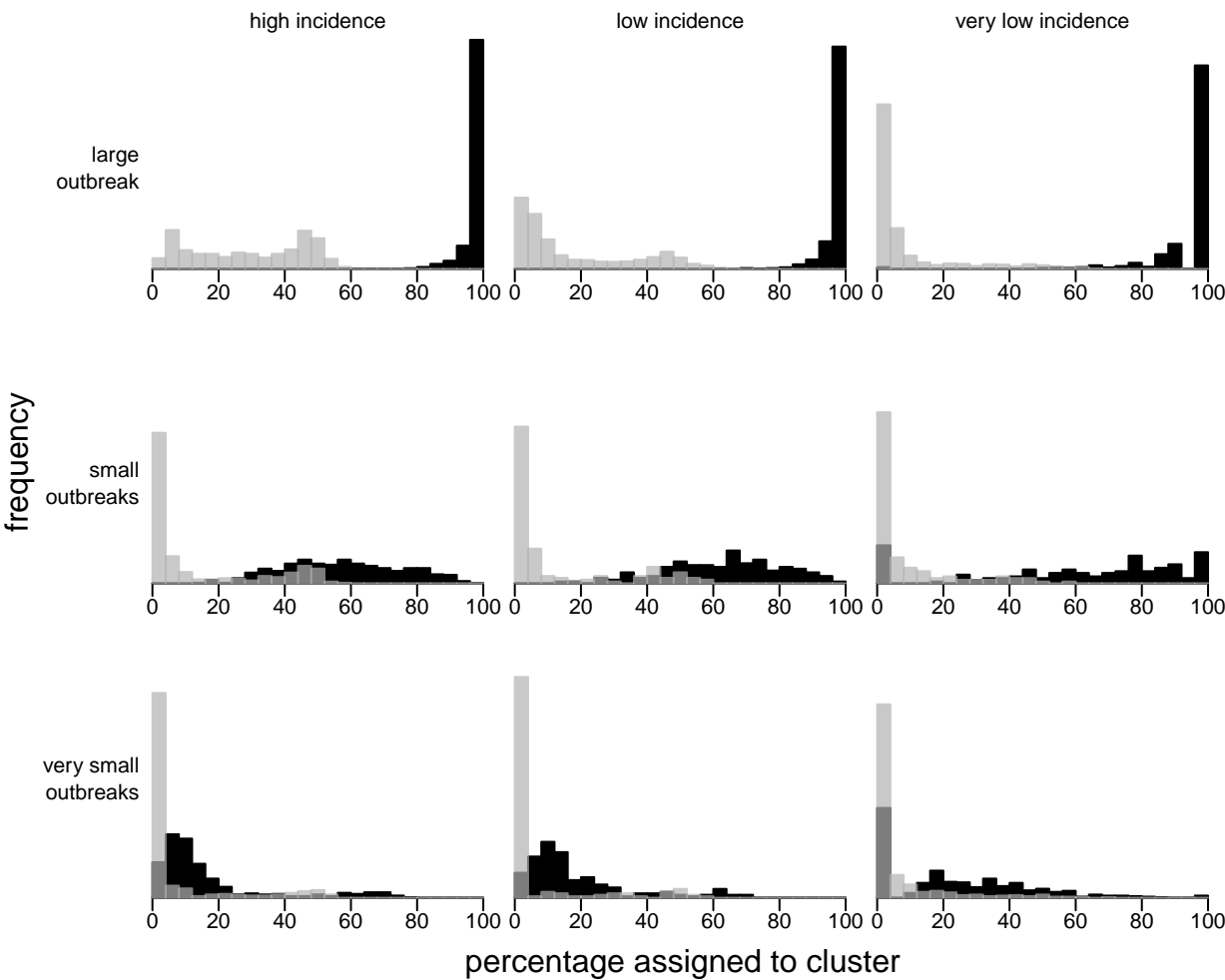

Supplement: Figure S3 — Sensitivity and false positive rate when 20% of cases are unobserved. Percentage of (black) outbreak and (gray) non-outbreak cases assigned to putative transmission clusters for simulations under nine different scenarios, when 20% of cases is unobserved. In each scenario, 10% of all cases is an outbreak case. Total expected number of cases is (left column) 1000, (middle column) 500 or (right column) 100. Outbreak cases belong to (top row) one large outbreak, (middle row) small outbreaks caused by 1/10 of cases being infectious with R = 0.5, (bottom row) minor outbreaks caused by all cases being infectious with R = 0.1. As expected, performance decreases when the distance between cases increases. A notable exception are the very small clusters, where sensitivity actually increases. As these transmission clusters are mainly of size two, discarding a case does not lead to larger distances, but to elimination of the cluster. Thus the number of cases and clusters is affected, but the intra-cluster distances are not. (PDF) [file pone.0069875.s004.pdf]
