# Supplementary material for: Finding Evidence for Local Transmission of Contagious Disease in Molecular Epidemiological Datasets
Source: PLoS One. 2013 Jul 26;8(7):e69875. doi: 10.1371/journal.pone.0069875 (PMC3724731; doi:10.1371/journal.pone.0069875)
Supplement: Table S1 — Median of sensitivity/false positive rate of assigning locally infected cases to a putative transmission cluster for simulated datasets where distances for infector-infected pairs are small. (DOCX) [file pone.0069875.s005.docx]

|  | High incidence | Low incidence | Very low incidence |
| --- | --- | --- | --- |
| Large outbreak | 1.00/0.22 | 1.00/0.01 | 1.00/0.00 |
| Small outbreaks | 0.68/0.01 | 0.71/0.00 | 0.71/0.00 |
| Very small outbreaks | 0.16/0.00 | 0.18/0.00 | 0.16/0.00 |
